# Endoscopic Submucosal Dissection for Colorectal Neoplasms: Is the West Catching Up?

**DOI:** 10.3390/cancers18142190

**Published:** 2026-07-08

**Authors:** Ishaan Vohra, Harishankar Gopakumar, Anuraga Meyyappan, Cody Chen, Garrett Blatter, Brian Martins, Shyam Thakkar, Neil Sharma

**Affiliations:** 1 Department of Medicine, University of Illinois College of Medicine Peoria, Peoria, IL 61605, USA; 2 Department of Gastroenterology, Carle Methodist Healthcare System, Peoria, IL 61636, USA; 3 Department of Gastroenterology, Brigham and Women’s Hospital, 75 Francis Street, Boston, MA 02115, USA; hgopakumar@bwh.harvard.edu; 4 Department of Medicine, American University of Antigua, University Park, Jabberwock Beach Road, Coolidge P.O. Box W1451, Antigua and Barbuda; anumey7@gmail.com; 5 Department of Medicine, University of Arizona College of Medicine-Phoenix, 475 N 5th St, Phoenix, AZ 85004, USA; cody.chen4@bannerhealth.com (C.C.); garrett.blatter5@bannerhealth.com (G.B.); brianmartins@arizona.edu (B.M.); 6 Division of Gastroenterology & Hepatology, Department of Medicine, West Virginia University, 5th Floor HSCS, Room 5500, Morgantown, WV 26506, USA; shyam.thakkar@hsc.wvu.edu; 7 Banner MD Anderson & BMG, Banner Health, 925 E. McDowell Rd, Phoenix, AZ 85006, USA; neil.sharma@bannerhealth.com

**Keywords:** endoscopic submucosal dissection, colorectal neoplasms, en bloc resection, Western practice, learning curve, training, artificial intelligence, guidelines

## Abstract

Endoscopic submucosal dissection (ESD) has revolutionized the management of superficial colorectal neoplasms, offering superior en bloc resection rates compared with conventional endoscopic mucosal resection (EMR). While ESD has been the standard of care in East Asian countries for over two decades, its adoption in Western countries has been considerably slower, hampered by the steep learning curve, prolonged procedural times, limited training infrastructure, and differences in disease epidemiology. However, recent years have witnessed a paradigm shift, with growing evidence from Western multicenter studies demonstrating outcomes that increasingly approach those reported from high-volume Eastern centers. The landmark RESECT-COLON randomized trial provided level-1 evidence supporting the superiority of ESD over piecemeal EMR for large colorectal polyps. Concurrently, novel training paradigms, technological innovations including traction-assisted devices and artificial intelligence (AI)-guided systems, and evolving societal guidelines from the American Gastroenterological Association (AGA), American Society for Gastrointestinal Endoscopy (ASGE), and European Society of Gastrointestinal Endoscopy (ESGE) are accelerating Western adoption. This state-of-the-art review comprehensively examines the current landscape of colorectal ESD in Western practice, highlighting the evolution of outcomes, training pathways, guideline recommendations, technological advances, and future directions. We provide a critical appraisal of the East–West outcome gap and discuss strategies to bridge this divide, positioning colorectal ESD as an increasingly viable first-line therapy for appropriate lesions in Western endoscopy centers.

## Simple Summary

Early colorectal cancers can often be treated without surgery using minimally invasive endoscopic techniques. Endoscopic submucosal dissection (ESD) is one such technique that enables the removal of superficial colorectal tumors in a single piece more often than conventional endoscopic mucosal resection (EMR). Although widely established in East Asia, where it was developed, its adoption in the West has been more gradual. This review evaluates the current state of Western practice, highlighting how recent clinical trials and updated gastroenterological society guidelines demonstrate growing adoption and outcomes approaching Eastern clinical benchmarks. However, widespread use of ESD in the West remains limited by a steep learning curve and a lack of structured training pathways. This review discusses these barriers and highlights future developments, including artificial intelligence, expanding clinical evidence, and formalized training curricula, that may further improve outcomes and support broader implementation.

## 1. Introduction

Colorectal cancer (CRC) remains the third most commonly diagnosed malignancy worldwide and the second leading cause of cancer-related mortality, accounting for approximately 1.9 million new cases and 930,000 deaths annually [1,2]. The global burden of CRC is disproportionately concentrated in Western countries owing to dietary and behavioral risk factors associated with industrialized lifestyles, though a rising incidence in developing nations undergoing economic transition has been well documented [3]. Screening colonoscopy programs have substantially improved early detection rates, leading to the identification of increasing numbers of superficial colorectal neoplasms amenable to endoscopic resection [4,5,6].

Endoscopic submucosal dissection (ESD) was initially developed in Japan in the late 1990s as an advanced technique for en bloc resection of early gastrointestinal neoplasms, particularly in the stomach where the prevalence of early gastric cancer provided ample opportunity for skill acquisition [7,8]. The technique was subsequently extended to the colorectum, with Japanese and Korean endoscopists achieving en bloc resection rates exceeding 90% and curative resection rates of 85–90% in large multicenter studies [9,10]. In contrast to conventional endoscopic mucosal resection (EMR), which frequently necessitates piecemeal removal of lesions larger than 20 mm, ESD enables precise circumferential incision and submucosal dissection regardless of lesion size, thereby providing intact specimens for accurate histopathological assessment and lower recurrence rates [11,12].

Despite these compelling advantages, the adoption of colorectal ESD in Western countries has been remarkably slow. Several interrelated factors have contributed to this disparity, including the steep learning curve, prolonged procedural times, higher complication rates during the training phase, the relative rarity of early gastric cancer in Western populations (which limits the step-up training pathway utilized in Asia), and the lack of standardized training and credentialing programs [13,14,15]. Furthermore, the well-established efficacy and safety profile of piecemeal EMR, combined with the application of snare-tip soft coagulation to resection margins, has provided Western endoscopists with a satisfactory, albeit imperfect, alternative [16,17].

However, the landscape of colorectal ESD in Western countries is undergoing a transformative shift. The past five years have seen a marked acceleration in adoption, driven by accumulating high-quality evidence, the establishment of structured training programs, technological innovations, and evolving guideline recommendations from major gastroenterological societies [18,19,20,21]. The publication of the RESECT-COLON trial in 2024, the first randomized comparative trial of ESD versus piecemeal EMR for large colorectal adenomas, provided landmark evidence demonstrating the superiority of ESD in achieving complete resection with lower recurrence rates [22]. While ESD has increasingly been adopted in Western practice for the management of gastrointestinal neoplasia, endoscopic full-thickness resection (EFTR) has emerged as a complementary resection strategy, particularly for difficult lesions in the colon and upper gastrointestinal tract that may not be amenable to conventional endoscopic techniques. The development of the full-thickness resection devices (FTRD^®^) has enhanced the safety and efficacy of EFTR as an alternative therapeutic approach for difficult lesions, such as non-lifting or fibrotic polyps, that might otherwise require surgery [23]. The development of EFTR highlights the rapid evolution of advanced endoscopic resection techniques in the West, where efforts to expand minimally invasive alternatives to surgery have accelerated. Within this evolving landscape, colorectal ESD remains a cornerstone technique whose adoption, implementation, and outcomes warrant a thorough evaluation. This review provides a comprehensive, state-of-the-art assessment of colorectal ESD in Western practice, examining outcomes, training paradigms, guidelines, technological innovations, and future directions.

## 2. Historical Evolution of Colorectal ESD

The development of ESD can be traced to the pioneering work of Hirao and colleagues in 1983, who first described a technique of local injection and incision for endoscopic resection of gastric lesions [24]. The technique evolved substantially through the 1990s with contributions from Gotoda, Ono, Saito, and other Japanese endoscopists who refined submucosal injection agents, electrosurgical knives, and dissection strategies [7,25]. The transition from gastric to colorectal ESD represented a significant technical challenge, as the thinner colonic wall, greater angulation, and inferior scope stability compared with the stomach increased the risk of perforation and prolonged procedural times [26].

Early case series from Japan in the early 2000s reported en bloc resection rates of approximately 80%, but with perforation rates exceeding 10% in some series, reflecting the inherent difficulty of the technique in the colorectum [27]. However, progressive refinements in technique—including optimization of submucosal injection agents, development of specialized electrosurgical knives (DualKnife, IT knife, FlushKnife), adoption of CO_2_ insufflation, and improved hemostasis and closure strategies—led to substantial improvements [28,29,30,31,32,33,34]. More recently, adjunctive strategies such as traction-assisted dissection—using double-clip, clip-band, or S-O clip techniques—and the pocket-creation method have further improved dissection efficiency and en bloc resection [35,36,37,38,39]. By the late 2000s and early 2010s, large Japanese multicenter prospective studies demonstrated en bloc resection rates of 88–95%, curative resection rates of 85–92%, and perforation rates that had declined to 2–5% [9,10,40].

The introduction of colorectal ESD to Western countries occurred gradually, beginning with early adopters in Europe (particularly France, Germany, the Netherlands, and the United Kingdom) and subsequently the United States [41,42]. Initial Western case series in the 2010s demonstrated considerably lower en bloc resection rates (70–85%) and higher complication rates compared with established Eastern centers, reflecting the early learning curve phase [43,44]. Nevertheless, these pioneering efforts established the foundation for the subsequent expansion of colorectal ESD across Western institutions.

## 3. Indications and Patient Selection

The appropriate selection of lesions for colorectal ESD is critical for achieving optimal outcomes and represents one of the most important determinants of procedural success. Current indications for colorectal ESD, as endorsed by major international guidelines, include laterally spreading tumors (LSTs) larger than 20 mm that are not amenable to en bloc EMR, lesions with suspected superficial submucosal invasion (Paris classification 0-Is, 0-IIa+IIc, 0-IIc), large nodular rectal lesions where accurate pathological staging is essential for treatment planning, and recurrent or residual lesions after prior endoscopic treatment with submucosal fibrosis [18,19,20,21].

The morphological classification of colorectal lesions using the Paris classification system, combined with advanced optical diagnosis using virtual chromoendoscopy (narrow-band imaging, blue-light imaging, linked-color imaging) and magnification endoscopy, plays a central role in risk stratification and treatment selection [45,46]. The JNET (Japan NBI Expert Team) classification and the NICE (NB-I International Colorectal Endoscopic) classification systems provide standardized frameworks for predicting the depth of submucosal invasion, thereby guiding the choice between endoscopic and surgical management [47,48].

Importantly, careful pre-procedural assessment is essential to avoid unnecessary ESD for lesions that could be adequately managed by EMR, as well as to identify lesions with deep submucosal invasion that would be better served by surgical resection. The ESGE 2024 updated guideline recommends ESD as the standard of care for large rectal lesions and suggests its consideration for large colonic lesions after careful endoscopic evaluation ruling out invasive cancer [18]. The AGA 2018 Clinical Practice Update on ESD in the United States endorsed ESD for colorectal lesions with suspected superficial submucosal invasion and for lesions that cannot be removed en bloc by conventional EMR [15]. A more recent 2025 AGA Clinical Practice Update further reinforced the role of endoscopic resection, including ESD, for T1 CRC, emphasizing the importance of en bloc resection for accurate pathological assessment [49].

## 4. Outcomes in Eastern Versus Western Centers

The comparative analysis of colorectal ESD outcomes between Eastern and Western centers provides critical insight into the current state of global adoption and the trajectory of Western practice. Table 1 summarizes the key outcome metrics from representative large-scale studies.

### 4.1. Eastern Center Outcomes

The most robust data on colorectal ESD outcomes originate from Japan, where national registries and large multicenter prospective studies have established benchmark standards. Saito et al. reported en bloc and curative resection rates of 88% and 89%, respectively, in a prospective multicenter cohort of 1111 colorectal tumors [9]. More recent data from the Japan Gastroenterological Endoscopy Society (JGES) registry have demonstrated further improvement, with en bloc resection rates exceeding 95% and perforation rates declining to below 3% in high-volume centers [40,55]. A 2022 study by Kobayashi et al. from a large prospective multicenter cohort confirmed these benchmarks, reporting en bloc resection rates of 96.3% with curative resection in 83.3% of cases and perforation rates of 7.4%, with no local recurrence over a median follow-up of 60 months [50].

### 4.2. Western Center Outcomes

Western colorectal ESD outcomes have improved substantially over the past decade. A landmark 2025 international multicenter study involving 547 consecutive colonic ESD procedures across 11 centers (8 US, 1 UK, 1 Italy, 1 Egypt) reported cancer in 12% of resected lesions, with non-curative resection in 59.1% of cancer cases and an overall adverse event rate of 8.8% [52]. This study highlighted that the majority of colonic ESDs in Western practice involve benign pathology, raising important questions about patient selection and appropriate utilization.

Data from high-volume Western centers have demonstrated outcomes approaching Eastern benchmarks. A retrospective analysis of 763 patients undergoing colorectal ESD at Brigham and Women’s Hospital between 2014 and 2024 reported en bloc resection in 95% of cases, R0 resection in 89%, and curative resection in 88%, with a mean procedure time of 64 min and delayed bleeding in 3% of cases [53]. Similarly, a 2025 prospective cohort study across 13 European centers involving 3770 colorectal ESDs demonstrated that high-volume centers achieved significantly better outcomes, including higher rates of complete and curative resections and faster procedures, compared with low- and medium-volume centers [51].

The RESECT-COLON trial, published in Annals of Internal Medicine in 2024, provided the first level-1 evidence comparing ESD with piecemeal EMR for large nonpedunculated colonic adenomas (>25 mm) [22]. Conducted across six French referral centers by 13 experienced endoscopists, the trial demonstrated that ESD achieved significantly higher en bloc resection rates with lower recurrence rates compared with EMR, albeit with a higher adverse event rate. This landmark trial validated the clinical benefit of colorectal ESD and provided a strong impetus for broader Western adoption.

A 2025 international multicenter study specifically examining the impact of prophylactic defect closure after colonic ESD found that closure significantly reduced delayed bleeding (1.7% vs. 5.6%, *p* = 0.03), supporting the adoption of routine defect closure as a strategy to mitigate post-ESD adverse events [56,57,58].

### 4.3. Bridging the East–West Gap

The differences in clinical outcomes between Eastern and Western centers appear to be influenced largely by procedural volume and operator experience [51,54]. Kobayashi et al. reported an en bloc resection rate of 96.3%, reflecting the maturity of colorectal ESD programs in Japan, where the procedure has been established for more than two decades and endoscopists maintain high annual volumes [50]. In contrast, the North American meta-analysis by Bowler et al., which included endoscopists of varying experience, reported a lower en bloc resection rate of 84.8%. Their findings highlighted variability in outcomes across contemporary North American ESD programs [54]. The variations in success and adverse event rates likely reflect, at least in part, the steep learning curve associated with Western adoption. While outcomes vary across North American centers, multiple learning-curve studies demonstrate improved outcomes with increased operator experience [59,60,61].

Notably the disparity in outcomes between Eastern and Western centers narrows considerably among high-volume Western ESD programs, underscoring the importance of procedural experience and institutional expertise. The Brigham and Women’s Hospital cohort reported a 95% en bloc resection rate, while a large European cohort, as described by Alfarone et al., demonstrated en bloc resection rates as high as 96% in high-volume centers [51,53]. Similarly, disparities in perforation and curative resections rates between Eastern and Western cohorts become less pronounced in mature Western ESD programs, with experienced centers achieving outcomes that closely approach those of Eastern cohorts [51,53].

Several distinct clinical and educational factors contribute to the remainder of the East–West disparity. First, Western referral centers frequently encounter previously manipulated lesions following attempted EMR, biopsy, or tattooing, resulting in submucosal fibrosis and more challenging dissections [53]. Second, the traditional Japanese training model, in which endoscopists gain proficiency in gastric ESD before transitioning to colorectal cases, is generally unfeasible in Western countries due to the lower prevalence of early gastric cancer [62]. Third, differences in lesion characteristics, including the higher proportion of right-sided colonic lesions with greater technical difficulty in Western series, may contribute to outcome disparities [52,63]. Taken together, these findings suggest that the East–West gap is narrowing, and that contemporary ESD outcomes are likely influenced by procedural volume, operator expertise, and lesion characteristics.

## 5. The Learning Curve: A Central Challenge

The learning curve for colorectal ESD represents the single most significant barrier to widespread adoption in Western countries. Unlike EMR, which can be learned within the framework of standard gastroenterology fellowship training, colorectal ESD requires mastery of multiple complex skills including precise circumferential mucosal incision, controlled submucosal dissection with appropriate tissue plane identification, management of intraprocedural bleeding, recognition and treatment of perforations, and knowledge of specialized electrosurgical devices and settings [64,65].

Learning curve analyses from both Eastern and Western centers have provided important benchmarks for skill acquisition. A seminal study by Jeon et al. demonstrated that endoscopists fully experienced in gastric ESD required approximately 50 colorectal ESD cases to achieve adequate proficiency, as measured by en bloc R0 resection rates exceeding 90% [66]. However, the experience of Western endoscopists, who typically lack prior gastric ESD experience, follows a different trajectory. A prospective single-center study from Poland demonstrated that after 76 procedures, en bloc resection rates surged to 86%, accompanied by a significant increase in resection speed to 9 cm^2^/h or greater, suggesting that Western endoscopists may require a longer learning curve [67].

A systematic review and meta-analysis presented at Digestive Disease Week (DDW) 2024 specifically examined the learning curve for colorectal ESD among endoscopists without prior ESD experience, providing pooled estimates that suggested 40–80 cases are needed to achieve competency depending on the outcome metric assessed [59]. A 2024 report on an untutored, single-operator learning curve from the United States demonstrated that satisfactory outcomes can be achieved through a prevalence-based approach with adequate case volume, though the initial phase is associated with higher complication rates and longer procedural times [60].

The concept of a “step-up” training approach has been advocated by several expert groups. Data from a German center demonstrated that a structured 12-month protocol involving 50 observational and supervised ESD cases plus 24 animal procedures yielded an R0 resection rate of 93% and a complication rate of 7% in the first 30 independent cases, primarily for rectal and gastric lesions [61]. However, this study also highlighted that ESD training in the proximal colon remains challenging even after initial competency is achieved, underscoring the anatomic complexity of right-sided colonic ESD.

## 6. Training Pathways in Western Countries

The establishment of effective training pathways for colorectal ESD in Western countries has emerged as a critical priority. Unlike Japan, where the master–apprentice model allows trainees to progress through a structured stepwise curriculum over several years, Western training has been characterized by heterogeneous approaches reflecting local expertise and resources [68,69,70].

### 6.1. United States

In the United States, ESD training has evolved through several parallel pathways. Advanced endoscopy fellowship programs have increasingly incorporated ESD training into their curricula, though the extent and quality of exposure remain variable [71]. Dedicated ESD courses, hands-on workshops, and live demonstrations at major academic centers have proliferated, providing introductory exposure to the technique [69,72]. Several high-volume centers, including Brigham and Women’s Hospital, AdventHealth Orlando, and the University of Florida, have emerged as de facto training hubs for colorectal ESD [53,60,73].

The AGA Clinical Practice Update on ESD in the United States, published in 2019 by Draganov et al., outlined a framework for ESD adoption including cognitive training, ex vivo and animal model practice, proctored human cases, and mentored independent practice [15,74]. However, a standardized national credentialing pathway remains absent, and the determination of competency is largely left to individual institutions.

### 6.2. Europe

European ESD training has been facilitated by the ESGE curriculum and several national ESD registries that provide standardized data collection and quality benchmarking [75,76]. France has emerged as a leader in colorectal ESD in Europe, benefiting from a collaborative network of high-volume referral centers that participated in the RESECT-COLON trial [22]. The Netherlands, Germany, and the United Kingdom have also developed structured ESD programs with centralized referral pathways [77,78,79]. The European Colorectal ESD Group has established prospective registries that enable tracking of procedural outcomes and learning curves across participating centers [51].

### 6.3. Stepwise Training Regimen

Given the limitations of human case volume in Western countries, ex vivo and simulation-based training have assumed an outsized importance. Ex vivo porcine models, including both gastric and colonic preparations, provide realistic tissue handling experience and have been validated as effective tools for ESD skill acquisition [80,81,82]. Gromski et al. demonstrated a clear learning curve for colonic ESD in an ex vivo simulator, with endoscopists achieving proficiency after approximately 30 procedures [78]. A proposed stepwise training regimen for Western endoscopists includes ex vivo gastric training, ex vivo colonic training, live porcine training, and finally mentored human training [69,80].

As Western trainees transition from animal models to supervised human cases, careful lesion selection is essential. Rectal lesions are generally favored for early clinical experience because they offer greater scope stability, a thicker muscularis propria compared with the colon, and improved maneuverability within a fixed pelvic location [61]. This approach is consistent with current guidance from ESGE which supports initial human ESD performance in the rectum or antrum [83]. In this context, a North American multicenter study of primarily novice ESD endoscopists reported an acceptable adverse event rate of 11.7% for rectal ESD, with no statistically significant difference relative to an experienced ESD endoscopist [84].

Table 2 summarizes the current training pathways available for colorectal ESD in Western countries.

## 7. Societal Guideline Recommendations

The endorsement of colorectal ESD by major gastroenterological societies has been instrumental in driving its adoption in Western practice. Over the past several years, guideline recommendations from both Eastern and Western societies have progressively expanded the recognized indications for ESD, although the strength of recommendations and quality of underlying evidence vary considerably across organizations. Table 3 summarizes the key guideline recommendations from major international societies in chronological order.

Notably, while the JGES and USMSTF issued strong recommendations as early as 2020, Western European and American societies have been more cautious, reflecting the limited availability of high-quality randomized evidence. The ESGE guidelines have progressively expanded indications for ESD, initially recommending it primarily for rectal lesions with suspected submucosal invasion (2022) and subsequently establishing it as standard of care for large rectal lesions (2024). The 2025 AGA Clinical Practice Update on T1 CRC represents a significant shift, explicitly endorsing en bloc resection—including ESD—as the preferred approach for suspected early colorectal cancers and defining curative criteria for endoscopic resection. The convergence of these guideline recommendations from diverse international societies underscores the growing global consensus supporting the role of colorectal ESD as a first-line therapeutic modality for appropriate lesions.

## 8. Persistent Challenges and Barriers to Adoption

Despite the encouraging trajectory of colorectal ESD adoption in Western countries, several significant challenges remain. First, the absence of standardized credentialing and privileging criteria for ESD across most Western healthcare systems creates uncertainty for both practitioners and institutions [15,69]. Unlike Japan, where the Japan Society of Gastroenterological Surgery has established clear competency benchmarks, Western societies have yet to define minimum case volumes, outcome thresholds, or proctoring requirements for independent ESD practice.

Second, reimbursement disparities persist. In many Western healthcare systems, ESD is reimbursed at rates comparable to EMR despite substantially greater procedural time, complexity, and resource utilization [85,86,87]. This financial disincentive may discourage centers from investing in the infrastructure and training necessary to develop ESD programs. Third, the concentration of ESD expertise in a relatively small number of academic referral centers raises concerns about geographic access and the referral burden on patients requiring ESD [51,88].

Fourth, the risk of unnecessary ESD—performing technically complex and resource-intensive procedures on lesions that could have been adequately managed by EMR—represents an important quality concern. The finding that the majority of colonic ESDs in a recent Western multicenter study involved benign pathology highlights the need for improved pre-procedural risk stratification and appropriate utilization frameworks [52]. Fifth, the management of complications, particularly delayed perforation in the right colon, requires institutional readiness and multidisciplinary collaboration with surgical teams, which may not be available at all centers [89].

## 9. Future Directions

The future of colorectal ESD in Western practice is likely to be shaped by several converging trends. The continued accumulation of high-quality evidence, including additional randomized controlled trials and prospective registries, will further define the role of ESD relative to EMR and surgery for specific lesion types and clinical scenarios. The integration of AI into ESD practice holds transformative potential [88,90,91,92]. AI-enhanced white-light colonoscopy systems have shown promise in differentiating superficial and deeply invasive CRC, which may improve patient selection for EMR, ESD, or surgical referral [93,94]. Similar deep-learning approaches have been validated for the detection of early neoplasia elsewhere in the gastrointestinal tract, supporting their broader applicability to ESD workflows [95,96]. Beyond pre-procedural assessment, emerging AI applications may support real-time intraoperative guidance, including recognition of submucosal vessels to reduce intraprocedural bleeding and prediction of mucosal incision guidelines [97,98]. As AI tools mature, they may help standardize lesion assessment and reduce outcome variability as ESD expands beyond expert referral centers [54].

The development of standardized, internationally recognized training curricula and competency assessments will be essential for ensuring safe, high-quality ESD practice as the procedure disseminates beyond expert referral centers. The creation of virtual reality simulation platforms with haptic feedback, combined with objective AI-based competency assessment tools, may accelerate the learning curve and provide scalable training solutions [94,99]. Robotic-assisted endoscopic platforms may ultimately address the fundamental ergonomic and technical limitations of manual ESD, potentially democratizing access to high-quality en bloc resection [100,101].

Finally, the development of risk prediction models incorporating clinical, endoscopic, and molecular biomarkers may enable increasingly precise patient selection for ESD, ensuring that the procedure is offered to those most likely to benefit while sparing others unnecessary procedural risk and resource utilization [102,103].

More recently, the emergence of endoscopic full-thickness resection (EFTR) using full-thickness resection devices (FTRD^®^) has offered additional therapeutic options for selected colorectal lesions, with early comparative studies demonstrating similar en bloc and R0 resection rates to ESD. A meta-analysis of four studies comparing 215 patients undergoing EFTR and 315 undergoing ESD found no significant difference between FTRD and ESD in en bloc resection (*p* = 0.31) or R0 rates (*p* = 0.42) for colorectal neoplasms. However, FTRD procedures were significantly quicker (mean time reduction, *p* = 0.004) and had fewer adverse events (*p* < 0.00001) [104]. Similarly, a multicenter randomized trial by Andrisani et al. directly compared FTRD vs. ESD for challenging colonic lesions (<30 mm), and found en bloc resection was achieved in 95.5% of FTRD cases vs. 93.3% of ESD cases, and R0 resection rates were statistically similar (93.3% vs. 80%; *p* = 0.06) [105]. Though ESD has expanded in the West for early GI cancers, future research will be needed to better define the optimal indications and relative roles of EFTR and ESD within advanced endoscopic practice.

## 10. Conclusions

Colorectal ESD has undergone a remarkable evolution from a niche East Asian technique to an increasingly accepted component of the therapeutic endoscopy armamentarium in Western countries. While the East–West gap in outcomes has not been fully eliminated, it has narrowed substantially, with high-volume Western centers now achieving en bloc resection rates, curative resection rates, and safety profiles that approach Eastern benchmarks. The RESECT-COLON trial has provided pivotal level-1 evidence supporting the clinical benefit of ESD over piecemeal EMR for large colorectal lesions.

The ongoing challenges of training, credentialing, reimbursement, and appropriate utilization require coordinated, multi-stakeholder solutions. The convergence of structured training programs, technological innovations, AI-guided procedural support, and evolving societal guidelines is creating a favorable environment for the continued expansion of colorectal ESD in Western practice. As the field matures, a collaborative, evidence-based approach that bridges the expertise of Eastern and Western endoscopy communities will be essential to optimizing patient outcomes and ensuring that the benefits of colorectal ESD are available to all patients who stand to gain from this transformative technique.

## Figures and Tables

**Table 1:** Comparison of Colorectal ESD Outcomes Between Eastern and Western Centers.

Study (Year)	Region	N	En Bloc (%)	R0/Curative (%)	Perforation (%)	Recurrence (%)
Saito et al. (2010) [9]	Japan	1111	88	89	4.9	0.7
Kobayashi et al. (2022) [50]	Japan	Multicenter	97.0	90.4	7.4	0
Alfarone et al. (2025) [51]	Europe	3770	91–96 *	82–90 *	2.5–5.8 *	<2
Karna et al. (2026) [52]	International	547	NR	Variable ^†^	8.8 (AE)	NR
BWH Cohort (2025) [53]	USA	763	95	88–89	0.8%	<2
Bowler et al. (2025) [54]	USA/Canada	1095	84.8	80/79	3.4	NR
Jacques et al. (2024) [22]	France (RCT)	Multicenter	High	Superior to EMR	Higher vs. EMR	Lower vs. EMR

* Range reflects low- to high-volume centers. ^†^ NCR was 59.1% for cancer cases. AE, adverse events; BWH, Brigham and Women’s Hospital; EMR, endoscopic mucosal resection; NR, not reported; RCT, randomized controlled trial.

**Table 2:** Training Pathways for Colorectal ESD in Western Countries.

Training Modality	Description	Advantages	Limitations
Ex vivo porcine models	Gastric and colonic tissue in simulator boxes	Realistic tissue handling; repeatable; low cost	Lacks in vivo conditions (bleeding, peristalsis)
Live porcine models	In vivo animal ESD with anesthesia support	Realistic physiologic conditions; complication management	High cost; logistical complexity; ethical considerations
Observational training	Observing expert ESD cases at high-volume centers	Cognitive skill development; technique exposure	No hands-on experience; limited availability
Proctored human cases	Performing ESD under direct expert supervision	Real clinical environment; immediate feedback	Requires expert availability; scheduling challenges
Hands-on workshops/courses	Structured courses at conferences (DDW, UEGW) or dedicated centers	Concentrated exposure; networking; expert faculty	Short duration; limited retention without follow-up practice
Advanced endoscopy fellowship	12-month fellowship incorporating ESD into curriculum	Longitudinal training; case volume accumulation	Variable ESD exposure across programs; not all offer ESD
Virtual reality/AI simulators	Computer-based simulation with haptic feedback	Unlimited repetition; objective assessment; emerging	Currently limited fidelity; not yet widely validated

ESD, endoscopic submucosal dissection.

**Table 3:** Summary of Societal Guideline Recommendations for Colorectal ESD.

Society	Year	Key Recommendation	Strength
AGA (Clinical Practice Update)	2019	ESD recommended for colorectal lesions with suspected superficial submucosal invasion; requires specialized training	Expert commentary
JGES	2020	ESD recommended for LSTs ≥ 20 mm, lesions with suspected submucosal invasion, and recurrent lesions	Strong recommendation
USMSTF	2020	En bloc resection preferred for suspected T1 CRC; referral to centers with ESD expertise recommended	Strong recommendation
ESGE (ESD Guideline)	2022	ESD should be considered for en bloc resection of colorectal (particularly rectal) lesions with suspected superficial submucosal invasion	Weak recommendation, low quality evidence
ESGE (Polypectomy Update)	2024	ESD is standard of care for large rectal lesions; may be considered for LNPCPs ≥ 20 mm in high-volume centers	Weak recommendation, low quality evidence
AGA (T1 CRC Update)	2025	En bloc resection (including ESD) preferred for suspected T1 CRC; curative criteria defined	Expert commentary

AGA, American Gastroenterological Association; CRC, colorectal cancer; ESD, endoscopic submucosal dissection; ESGE, European Society of Gastrointestinal Endoscopy; JGES, Japan Gastroenterological Endoscopy Society; LNPCPs, large nonpedunculated colorectal polyps; LSTs, laterally spreading tumors; T1, tumor invading the submucosa; USMSTF, United States Multi-Society Task Force on Colorectal Cancer.

## Data Availability

No new data were created or analyzed in this study. Data sharing is not applicable to this article.
